# The President’s Address to the Members Texas State Medical Association

**Published:** 1889-02

**Authors:** 


### EDITORIAL DEPARTMENT.

F. E. DANIEL, M. D., EDITOR AND PUBLISHER.

THIS JOURNAL, by unanimous vote of the members, was made the Official Organ of the Austin District Medical Society.

COLLABORATORS:

*Dr. R. M. Swearingen, Austin.*
*Prof. B. E. Hadra, M. D., Galveston.*
*Prof. Geo. Cupples, M. D., San Antonio.*
*Dr. T. C. Osborn, Cleburne, Texas.*
*Dr E. J. Doering, Chicago.*
*Dr. E. J. Beall, Fort Worth.*
*Dr Odo Betz, Germany.*
*Dr. J. W. McLaughlin, Austin.*
*Dr. T. J. Tyner, Austin.*
*Prof. J. F. Y. Paine, M. D., Galveston.*
*Dr. R. H. L. Bibb, Mexico.*
*Dr. T. J. Bennett. Austin.*
*Dr. Bat Smith, Wharton.*
*Dr. E. Meierhof, New York.*
*Prof. W. B. Rogers, M. D., Memphis, Tenn.*

### THE PRESIDENT'S ADDRESS TO THE MEMBERS TEXAS STATE MEDICAL ASSOCIATION.—A CRISIS.

The President's address to the members of the Texas State Medical Association, which appears in this issue of the JOURNAL, and which has been sent out in a circular letter to the profession, was delayed some ten days or longer in the hands of the secretary, in consequence of the rush of business in all the printing establishments in Austin. The legislature is in session, and is turning out work by the ream; and although the State printer and the society's printers are running double force, night and day, it is well nigh impossible to get any job work done. The delay, we trust, is no serious matter; and the fault, if any be found, is chargeable to the secretary, and not to our worthy president.

We commend the address to the careful perusal of members, and we hope they will heed its admonitions, and laying aside all else, for four days, go to San Antonio and aid, by their presence and counsel, the efforts being made by the zealous President and a few stand-bys, to make the meeting a big success, in every sense of the word. A crisis exists in the life of the society. We intimated in our last that the District societies are becoming rivas, and in a measure jeopardizing the integrity of the central organization. It will be observed that the same fear has suggested itself to President Paine, and he sounds a note of warning.

The friends of the old asssciation, an organization which has done noble work, must rally to its defense. It has redeemed the profession of Texas from the false position, one of insignificance, to say the least, it so long occupied in the eyes of the outside world, and given it honor and character, a position second to none in America. District societies are very good and desirable, and should be encouraged, but they can never accomplish the work of a thorough regeneration of the State in the matters of public sanitation; can never hope or expect, as can a State organization, to become, as it should, the medical department of the State government, as the Alabama State Medical association is in that State. Its constitution is a part of the organic law of Alabama; it creates and operates the State board of health, which administers quarantine, records vital statistics, supervises the sale of food, drugs, liquors, etc., and suggests, if not dictates, sanitary legislation; it regulates the practice of medicine by examining applicants for the privilege of practicing in Alabama, licencing the competent and rejecting the incompetent. Many of these latter and their kind from other states come to Texas. The same work which has been done, and is now being done, in Alabama and other States, remains to be done in Texas, and it is the mission of the State Medical Association to do it. It were preposterous to suppose that such work could be done without a hearty co-operation of the local societies with the State association, in which they should be represented by delegates; and yet, practically, these local societies are, in a sense, its rivals; by satisfying the selfish ambition of some members, they are induced to give up their membership in the State Association. This should not be, and we trust the thoughtful members will use their influence to secure a large attendance. One more full, rousing meeting, such as was held at Belton in 1884, would insure renewed interest and strength, and an indefinite lease of life for the grand old mother society.

---

DR. JOHN PRESTON, was, by the board of managers unanimously re-appointed first assistant physician to the State Lunatic Asylum, notwithstanding a reported effort to trade him off.

